# Direct enhancement of nitrogen-15 targets at high-field by fast ADAPT-SABRE

**DOI:** 10.1016/j.jmr.2017.10.006

**Published:** 2017-12

**Authors:** Soumya S. Roy, Gabriele Stevanato, Peter J. Rayner, Simon B. Duckett

**Affiliations:** a Department of Chemistry, University of York, Heslington, YO10 5DD York, United Kingdom; b Institut des Sciences et Ingénierie Chimiques, Ecole Polytechnique Fédérale de Lausanne (EPFL), Lausanne 1015, Switzerland

**Keywords:** Hyperpolarization, PHIP, SABRE, ADAPT

## Abstract

Signal Amplification by Reversible Exchange (SABRE) is an attractive nuclear spin hyperpolarization technique capable of huge sensitivity enhancement in nuclear magnetic resonance (NMR) detection. The resonance condition of SABRE hyperpolarization depends on coherent spin mixing, which can be achieved naturally at a low magnetic field. The optimum transfer field to spin-1/2 heteronuclei is technically demanding, as it requires field strengths weaker than the earth’s magnetic field for efficient spin mixing. In this paper, we illustrate an approach to achieve strong ^15^N SABRE hyperpolarization at high magnetic field by a radio frequency (RF) driven coherent transfer mechanism based on alternate pulsing and delay to achieve polarization transfer. The presented scheme is found to be highly robust and much faster than existing related methods, producing ∼3 orders of magnitude ^15^N signal enhancement within 2 s of RF pulsing.

## Highlights

• SABRE hyperpolarization is achieved at high field by the ADAPT pulse sequence.
• A theoretical description of ADAPT-SABRE is linked to related methods for comparison.
• ADAPT-SABRE achieves faster magnetization transfer than several analogous approaches.
• ∼3 orders of magnitude signal enhancement produced in 1.6 s for a ^15^N target.

## Graphical abstract

**Figure:**
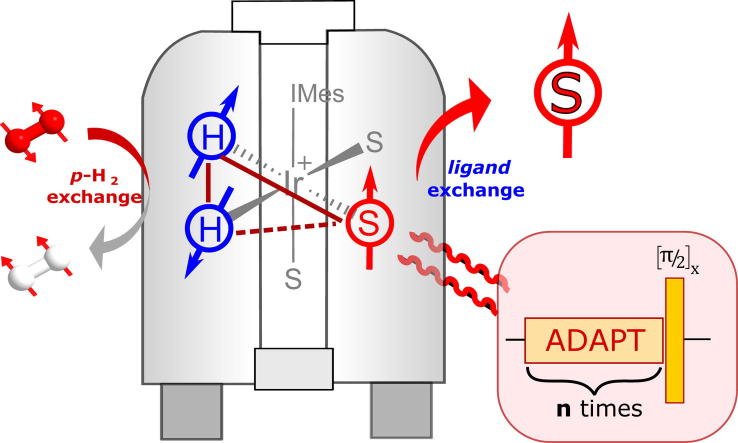

## 1 Introduction

Despite the huge success of NMR in a wide assortment of research fields ranging from structural material characterization to the imaging of internal human organs, it is still regarded to be underexploited based on its theoretical potential [1], [2]. Most of the successes of NMR and MRI applications have been achieved utilizing the thermal level of nuclear spin polarization which is only of the order of 10^−5^ at room temperature in a standard high field spectrometer [2]: only one spin in 30,000 contributes to the NMR signal for protons in a 9.4 T magnet. Improving this poor sensitivity would make NMR and MRI more widespread and cost-efficient. The solution to this challenge is offered by hyperpolarization methods that enhance the nuclear spin polarization by up to 5 orders of magnitude compared to standard thermal polarization [3]. This large sensitivity enhancement enables the completion of high-end MRI applications e.g. *in vivo* study of human cancer, which could otherwise not be performed due to sensitivity issues [4], [5], [6].

**Fig. 1:**
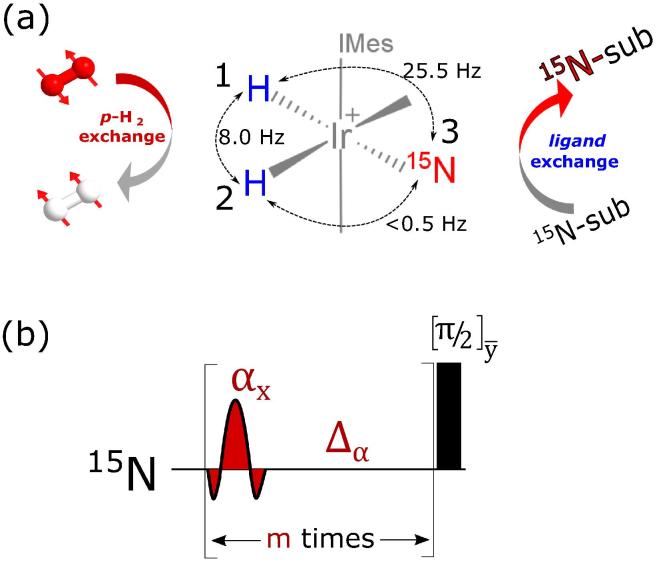
(a) Schematic depiction of SABRE hyperpolarization mechanism. The standard Iridium based metal catalyst (Ir-Imes) is used with IMes = 1,3-bis(2,4,6-trimethylphenyl) imidazol-2-ylidene. Singlet order from *para*-hydrogen is introduced reversibly to the hydrides, which subsequently transfer the polarization to substrate spins (^15^N-sub) via a J-coupling network under suitable resonance conditions. (b) ADAPT pulse sequence used in this study to achieve high-field SABRE transfer.

Within the class of hyperpolarization techniques, the Para-hydrogen Induced Polarization (PHIP) method employs a highly ordered nuclear singlet, para-hydrogen (*p*-H_2_) gas, to enhance poorly polarized substrate spins by several orders of magnitude [7], [8]. An important variant of the PHIP technique, SABRE was introduced in 2009, that no longer requires active hydrogenation to hyperpolarize targeted molecules [9]. It relies on the temporary association of the substrate at a metal center where the associated J-coupling network ultimately enables the generation of hyperpolarized substrates (see Fig. 1a). SABRE provides a simple, fast and cost-efficient approach to hyperpolarize substrates in its original form and they can be re-polarized several times in quick succession, providing the opportunity to achieve continuous hyperpolarization [10]. The method has been successful in polarizing a large class of biologically relevant substrates where their ^1^H, ^13^C and ^15^N nuclei [11], [12], [13], [14] are senitised, and also in preparing long-lived forms that are detectable for up to 30 min [15], [16], [17], [18], [19]. Considering their biological relevance, hyperpolarizing spin-1/2 heteronuclei already show great importance for *in vivo* MRI studies [20], [21].

The coherent spin mixing condition for heteronuclei in SABRE can be achieved by bubbling the solution at ultra-low magnetic field (typically 0.2–1.0 μT), as previously shown by Theis et al. [22]. However, this low-field technique requires field-cycling between low and high magnetic fields, a condition that is both technically demanding and unsuitable for immediate signal detection. Overcoming this challenge, Warren and co-workers proposed the LIGHT-SABRE approach to create a similar resonance condition at high field by applying an optimized spin-lock based RF pulse sequence [23]. Pravdivtsev et al. have since developed a ramp based CW pulse to achieve hyperolarization in heteronuclei [24], [25].

Here we show that, the recently published ADAPT (Alternating Delays Achieve Polarization Transfer) sequence [26] can be applied to transfer polarization from singlet hydrides to target ^15^N nuclei in a repetitive fashion. It takes only few seconds to build up strong ^15^N hyerpolarized signal whilst the transfer mechanism is fully compatible with LIGHT-SABRE [23], Level Anti-Crossing (LAC) [27] and PHIP spin-order transfer mechanisms [28].

This article is organized as follows: In Section 2 we briefly describe the ADAPT method and its optimizations by simulations to yield a SABRE perspective. In Section 3 we present experimental details and results. Finally, Section 4 contains conclusions and discussions.

## 2 Methods

### 2.1 ADAPT approach

We consider a system formed by three nuclear spins: two hydride ^1^H (**1** and **2** in Fig. 1a) and a ^15^N (**3** in Fig. 1a). The aim is to transfer nuclear spin polarization between the ^1^H singlet spin population and ^15^N longitudinal magnetization. ^1^H chemical equivalence is assumed, and it is experimentally imposed by CW irradiation on the ^1^H channel.

In a doubly rotating frame, if the ^1^H nuclei are chemically equivalent, the J coupling terms form the coherent Hamiltonian:

(1) $$H_{J}=\omega_{J}I_{1}\cdot I_{2}+\frac{\omega_{\Sigma }+\omega_{\Delta }}{2}I_{1z}I_{3z}+\frac{\omega_{\Sigma }-\omega_{\Delta }}{2}I_{2z}I_{3z}$$

where $\omega_{J}=2\pi J_{12}\text{,}\omega_{\Sigma }=2\pi (J_{13}+J_{23})$ and $\omega_{\Delta }=2\pi (J_{13}-J_{23})$. We use a basis formed by the direct product of the singlet-triplet basis for ^1^H spins **1** and **2**, and the eigenbasis for the $I_{3x}$ operator of ^15^N spin **3**. In this basis, the Hamiltonian in Eq. (1) can be decomposed into the direct sum of four orthogonal subspaces as detailed in Ref. [26]. However, we restrict the ADAPT analysis to the two relevant orthogonal subspaces $H^{a\text{,}b}$ and $H^{c\text{,}d}$ containing the proton singlet state:(2)(3)where $\left|a\rangle =-(|S^{0}\text{,}\alpha \rangle -|S^{0}\text{,}\beta \rangle )\text{,}|b\rangle =(|T^{0}\text{,}\alpha \rangle +|T^{0}\text{,}\beta \rangle )\text{,}|c\rangle =-(|T^{0}\text{,}\alpha \rangle -|T^{0}\text{,}\beta \rangle \right)$ and $\left|d\rangle =(|S^{0}\text{,}\alpha \rangle +|S^{0}\text{,}\beta \rangle \right)$. The out-of-diagonal elements $-\frac{\omega_{\Delta }}{4}$ in Eqs. (2), (3) can be exploited to transfer polarization from $\left|S^{0}\rangle \langle S^{0}\right|$ to $I_{3x}$. One of such methods is the ADAPT sequence as shown in Fig. 1b. It performs the task via a number of RF pulses and delays that are recursively repeated. Small and/or large α tip angle pulses can be used, providing the experimentalist a further degree of freedom. The protocol includes a number of steps: (i) definition of the tip angle: for example α=30°, (ii) calculation of the conversion efficiency for a given range of delays Δ and loop numbers *m* as detailed in [26], (iii) choice of the optimal pair $\Delta_{\mathrm{opt}}$ and $m_{\mathrm{opt}}$. A 90° pulse at the end of the loops (see Fig. 1b), is inserted to transform $I_{3x}$ into $I_{3z}$ so as to retain magnetization on the substrate upon dissociation. For the present case, the J-coupling network is known: $J_{12}=8$ Hz, $J_{13}-J_{23}=25.5$ Hz, and $J_{13}+J_{23}=25.5$ Hz and a theoretical transfer of 93% is achieved by ADAPT_30_ in about 40 ms with Δ=8 ms and m=5. In practice, multiple repetitions of this block are required to build up bulk longitudinal heteronuclear magnetization (see Fig. 5). To keep our studies simple, in this work we employ ADAPT_30_ which experimentally we found to be optimal, however as predicted all of the combinations of α,Δ and *m* we tested produced a return [26].

ADAPT remains robust even in the case of uncertainty about the value of the heteronuclear J_23_ (simulations not shown).

### 2.2 Initial state and trajectories

When the *para*-hydrogen and the substrate bind to the catalyst, the initial state can be represented as an overpopulation of the ^1^H scalar singlet order. We can define a set of orthogonal operators $I_{x}^{\mathrm{rs}}\text{,}I_{y}^{\mathrm{rs}}\text{,}I_{z}^{\mathrm{rs}}$ with cyclic commutation relationships $[I_{x}^{\mathrm{rs}}\text{,}I_{y}^{\mathrm{rs}}]={\mathrm{iI}}_{z}^{\mathrm{rs}}$, for each of the 2×2 subspaces in Eqs. (2), (3). The initial state and the observable $I_{3x}$ ^15^N-transverse magnetization are related to the following single transitions operators:

(4) $$\begin{array}{cc} & \left|S_{0}\rangle \langle S_{0}|\propto \frac{1}{2}(I_{z}^{\mathrm{ab}}-I_{z}^{\mathrm{cd}}\right)\\ & \frac{I_{3x}}{2}\propto -\frac{1}{2}(I_{z}^{\mathrm{ab}}+I_{z}^{\mathrm{cd}}) \end{array}$$

**Fig. 2:**
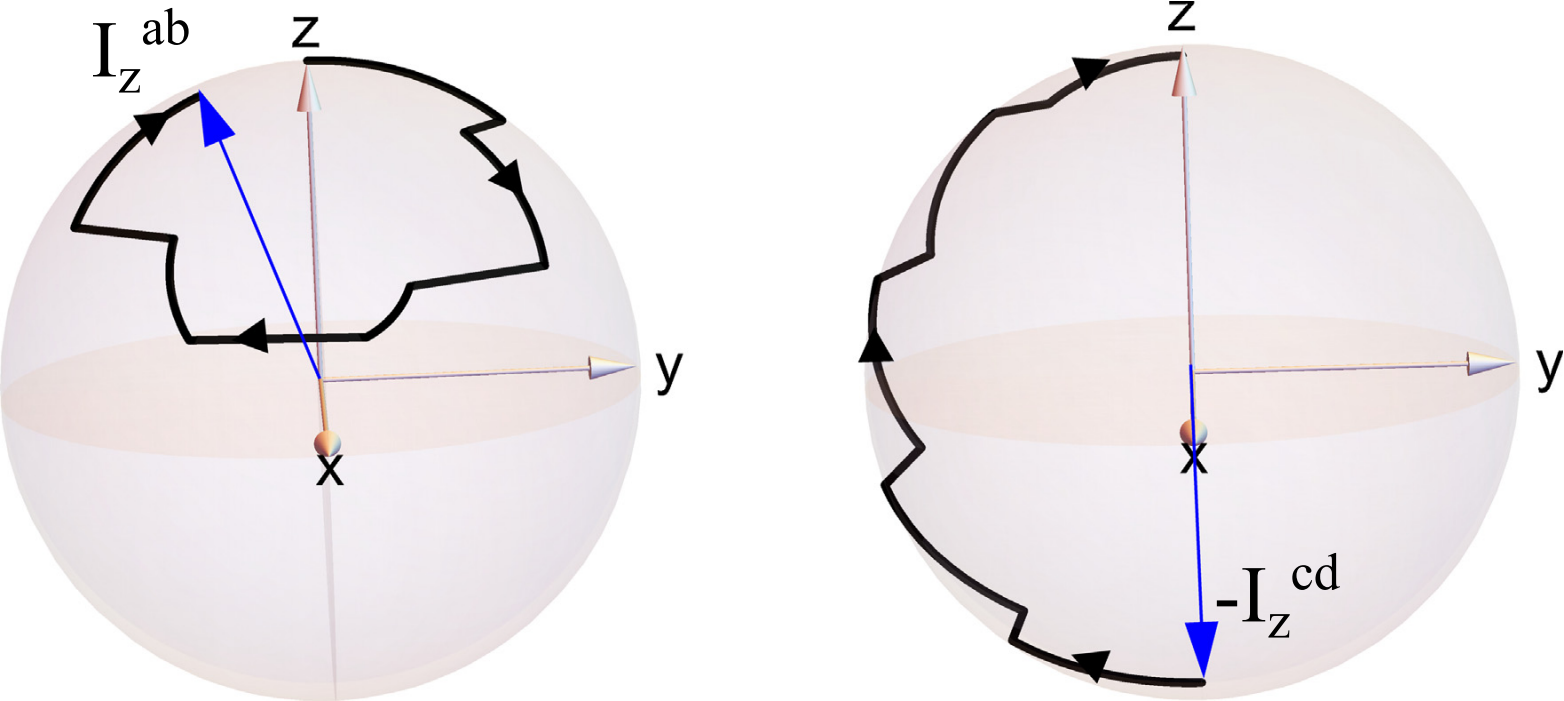
ADAPT induced rotations in the subspaces $H^{a\text{,}b}$ and $H^{c\text{,}d}$ upon application of ADAPT_30_ for Δ=8 ms and m=5 for J_12_ = 8 Hz, J_13_-J_23_ = 25.5 Hz.

From the set of Eq. (4), it is apparent that polarization transfer can be obtained, for example, when a sequence of events invert the sign of the operator $I_{z}^{\mathrm{cd}}$ while maintaining the sign of the operator $I_{z}^{\mathrm{ab}}$. ADAPT achieves this by performing a π rotation in the subspace $H^{c\text{,}d}$ (see Fig. 2).

### 2.3 Analogy with the LIGHT-SABRE and the LAC approach

A LAC [27] occurs when (i) the energy level relative to a pair of states |m〉 and |n〉 is equal $E_{|m\rangle }=E_{|n\rangle }$ and (ii) there is a matrix element $V_{\mathrm{mn}}=\langle m|V|n\rangle$ for some operator *V* that splits them. Essentially, the LIGHT-SABRE and LAC methods share the same clever idea: a resonance condition between the spin populations of the subspaces $H^{a\text{,}b}$ and $H^{c\text{,}d}$ can be established upon spin locking on the heteronuclear channel. The Hamiltonian operator for a constant RF heteronuclear irradiation is $H_{\mathrm{RF}}=2\pi \nu_{\mathrm{RF}}I_{3x}$. The ramp modulation used in the LAC method can be included by introducing a time dependency in the term $\nu_{\mathrm{RF}}$. For simplicity, here we disregard the time dependency $\nu_{\mathrm{RF}}$ as in the LIGHT-SABRE protocol [23]. The matrix representation of the Hamiltonian operator $\overset{∼}{H}=H_{J}+H_{\mathrm{RF}}$ transforms Eqs. (2), (3)into:(5)(6)

In Eqs. (5), (6), when $\nu_{\mathrm{RF}}=J_{12}\text{,}E_{|a\rangle }=E_{|b\rangle }=E_{|c\rangle }=E_{|d\rangle }$ so that a level crossing occurs. More importantly, in presence of magnetic inequivalence $\omega_{\Delta }\;\neq \;0$, and a LAC can be established for each subspace in Eqs. (5), (6). As a result, the heteronuclear J-coupling imbalance removes the degeneracy and promotes spin population transfer in each subspace ${\overset{∼}{H}}^{a\text{,}b}$ and ${\overset{∼}{H}}^{c\text{,}d}$. The term $\omega_{\Delta }$ is completely analogous to $V_{\mathrm{mn}}$. The duration of the optimal spin lock is inversely proportional to $\sqrt{2}\omega_{\Delta }$ [23]. To complete the analogy with the ADAPT method, it has also been noted by Theis et al. [23], that the effect of the CW irradiation is to produce a π pulse which transfers population between the hydrogen singlet population to the ^15^N transverse magnetization: precisely the same transformation achieved by ADAPT in the subspace of Eq. (3)(see Fig. 2).

## 3 Experimental results and discussions

**Fig. 3:**
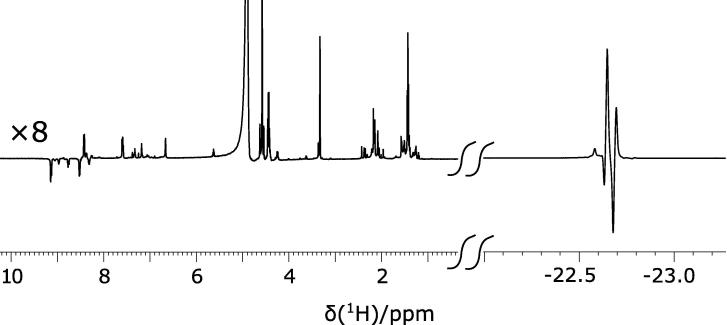
^1^H PHIP NMR spectrum of ^15^N- ethylnicotinate measured in a 500 MHz spectrometer, showing enhanced hydride region. The coupling constants presented in Fig. 1a can be calculated from the spectrum. The ‘down-field’ region (0–10 ppm) is vertically multiplied by 8 compared to the ‘up-field’ (hydride) region.

All the measurements were carried out with a 500 MHz Bruker Avance III spectrometer equipped with a broad-band (BBO) probe at 298 K. The sample was prepared in a 5 mm NMR tube by mixing 10 mM of [IrCl(COD)(IMes)] (IMes = 1,3-bis(2,4,6-trimethylphenyl) imidazole-2-ylidene, COD = cycloocta-1,5-diene) pre-catalyst and 50 mM of ^15^N-ethylnicotinate [29] in 0.6 ml methanol-$d_{4}$ solution. A valve-controlled *para*-hydrogen flow PTFE (polytetrafluoroethylene) tube was immersed inside the NMR tube to bubble the solution with *para*-hydrogen originating from a para-hydrogen generator with 90% enrichment. To accurately assign the resonances of the concerned spins and measuring their coupling constants within the network, we first performed a standard in-magnet PHIP experiment by bubbling *p*-H_2_ inside the spectrometer and recording a ^1^H spectrum upon applying a 45° pulse on the proton channel. A large and transient antiphase spectrum is observed in the hydride region reflecting the hydrogenation product, resulting from reaction with *p*-H_2_. Both the hydrides (spin-**1** and -**2**) show overlapping resonances at −22.66 ppm as shown in Fig. 3. The ^2^J_*HH*_ and *cis*-^2^J_*NH*_ coupling constants were simply measured from the hydride region of the spectrum. Whilst *trans*-^2^J_*NH*_ could not be observed it is expected to be negligible (<0.5 Hz) in these types of system [12], [27].

Next we performed a standard SABRE-SHEATH experiment [22] by shaking the solution with *p*-H_2_ inside a μ-metal can and then quickly recording a ^15^N spectra upon a 90° detection pulse. The purpose of this experiment was to find out the ^15^N resonance frequencies for the different forms of the substrate; the substrate can be *free* from the catalyst and also *bound* to the catalyst. Two different forms of *bound* substrate exist here (equatorial and axial position). In our study, we observe ^15^N *free* resonance at 303.22 ppm and equatorial-*bound* resonance at 256.60 ppm whilst the axial-*bound* resonance remains undetected due to insufficient detectable polarization. The parameters obtained from these initial screening experiments were sufficient to optimize the ADAPT pulse sequence numerically (see Fig. 1b).

**Fig. 4:**
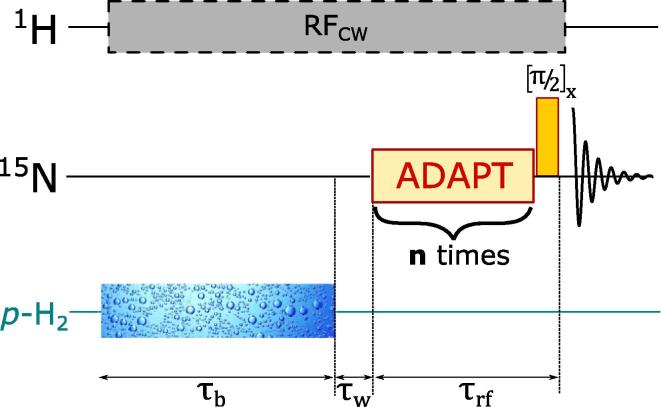
Experimental timings and RF sequence for the high-field ADAPT-SABRE experiment. The solution was bubbled by p-H_2_ gas for the duration of $\tau_{b}$; after that an appropriate waiting time ($\tau_{w}$) was provided to settle down the solution before applying the ADAPT pulse sequence (as shown in Fig. 1b) in ^15^N channel on-resonance and selectively exciting the equatorial *bound* peak. The ADAPT block was repeated n times before a final 90° hard pulse detects the signal. A low-powered continuous wave (CW) pulse was applied on ^1^H channel throughout the experiment on-resonance to the hydrides region.

The high-field SABRE experiments were performed according to the experimental protocol depicted in Fig. 4. After a suitable relaxation delay, *p*-H_2_ was bubbled through the solution for a duration of $\tau_{b}$ (typically 10 s) followed by a short waiting period ($\tau_{w}$ ∼ 1 s.) for the solution to settle. The optimized ADAPT pulse sequence immediately followed with a total duration of $\tau_{\mathrm{rf}}$ (≈Δ×m×n), selectively exciting the *bound*-^15^N resonance without affecting the *free* resonance. The offset for ^15^N channel was set on the *bound*-peak (256.60 ppm) whilst the band-width (BW) of the RF pulse was kept at 500 Hz (≈10 ppm). After *n* number of repeated ADAPT blocks, a hard 90° pulse was applied to detect the magnetization. During the RF sequence, a continuous-wave (CW) pulse of 1 kHz band-width was applied in proton channel resonant with the hydride region to enforce chemical equivalence between the hydride protons. However, when performed without any CW pulse in proton channel, we observe polarization transfer, albeit with a 30–40% of less enhancement than earlier.

**Fig. 5:**
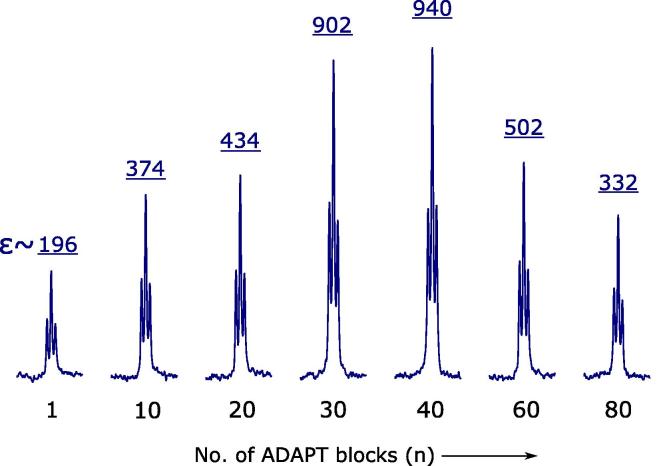
ADAPT-SABRE derived ^15^N spectra showing *free* substrate peaks with increasing numbers (n) of ADAPT block (see Fig. 4). A maximum enhancement of 940-fold was achieved at n = 40.

Using the numerically optimized parameters (see Section 2), the ADAPT sequence efficiently transfers singlet-order polarization of hydrides into longitudinal polarization of ^15^N nuclei that are connected to the network. An optimum level of polarization can be realized for ADAPT_30_ with Δ = 8 ms and m=5 in a total pulse duration of only 40 ms. Using these parameters, one ADAPT block achieves 196-fold enhancement factor (∊) for the *free* ^15^N target. The enhancement factor (∊) of hyerpolarization was calculated as the ratio of the integral for the hyperpolarized signal and the integral for the thermal signal divided by the number of thermal scans. As SABRE is an exchangeable process, the polarization transfer rate is limited by the residence time of both the hydrides and the free substrate, which are in the range of 100–200 ms under standard SABRE conditions. As a result, hydrogenation with *p*-H_2_ and therefore the polarization transfer does not occur simultaneously across the entire sample volume. For this reason, the ADAPT block has to be repeated multiple times to build up ^15^N polarization. In practice, one block of ADAPT sequence transfers only a fraction of available polarization into ^15^N magnetization. But as long as fresh *p*-H_2_ keeps exchanging together with non-polarized substrates, we observe accumulation in ^15^N magnetization by repeated application of the ADAPT block. Fig. 5 presents a gradual build-up in *free* ^15^N magnetization with increasing numbers of ADAPT blocks. A maximum enhancement of 940-fold was achieved after 40 ADAPT blocks of total duration ∼1.6 s. Ultimately, spin relaxation together with *p*-H_2_ consumption take over in the magnetization build-up process and ∊ starts decreasing with larger loop counts. The ∊ is also limited by several other factors e.g. RF inhomogeneity and imperfections caused by mixing and diffusion [30], [31].

**Fig. 6:**
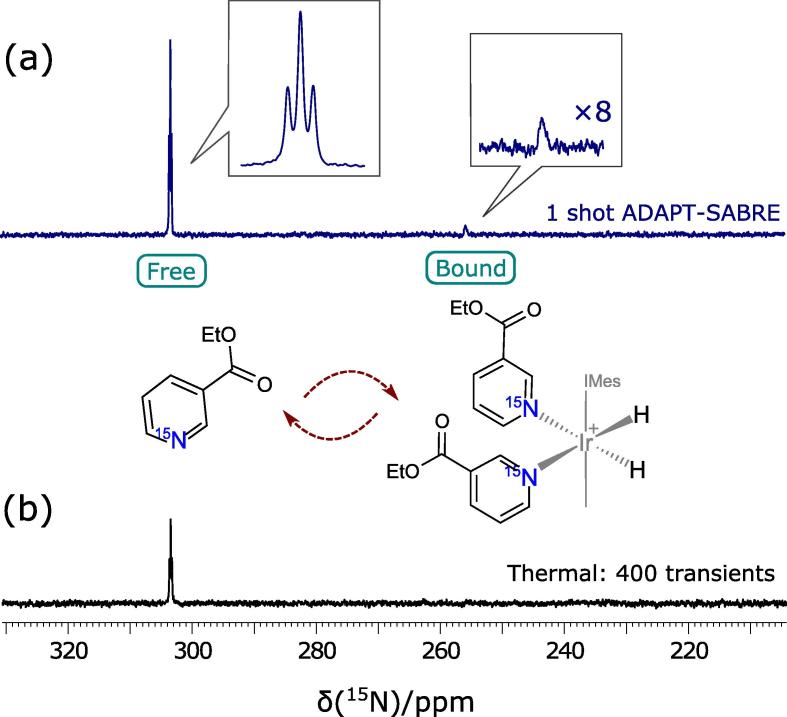
^15^N NMR spectra of ^15^N-ethyl nicotinate showing hyperpolarized spectra acquired by ADAPT-SABRE method in seconds and (b) corresponding thermal spectra obtained by 90° hard pulses over 400 transients with 120 s of recycle delays (total experimental time of 13.5 h). The *free*^15^N resonance peak was observed at 303.2 ppm whilst the ‘equatorial-bound’ peak was found at 255.6 ppm.

In Fig. 6 we show the success of the ADAPT-SABRE method in driving polarization from *singlet*-hydrides to *bound* ^15^N nuclei of ethylnicotinate and enhancing the *free* ^15^N response. The earlier parameters were used to achieve the 1-shot ADAPT-SABRE spectra in Fig. 6a. The corresponding thermal signal in Fig. 6b was acquired by averaging 400 transients with a recycle delay of 120 s (T_1_(^15^N) = 21.5 ± 0.5 s), taking over 13.5 h. The *bound* peaks remain undetectable in the thermal measurements even after many scans signifying greater relative enhancements as predicted previously [25]. The lack of a thermal signal for the bound peaks can be attributed to their broad line shapes, they are visible after 2000 scans.

## 4 Conclusions

In summary, we have used ADAPT-SABRE to generate ^15^N hyperpolarization at high magnetic field without the requirement of below-earth field sample mixing. The conversion is robust and faster than previously reported methods: it took only 1.6 s to reach nearly 3 orders of signal enhancement for a ^15^N target. This method has several advantages over the low-field SABRE mechanism, e.g. constant field shuttling, unnecessary signal losses during transport. The presented scheme whilst being inherently simple, can be easily augmented to any SABRE active species and their ^13^C, ^19^F and ^31^P nuclei. We are currently working on its further optimization via exchange rate, sample concentration and additive-dependence studies. In the future, we plan to examine how the dynamics of SABRE exchange can be harnessed to improve this ADAPT process. We believe it will be of particular importance in terms of achieving *in vivo* hyperpolarization, where sample transport poses a significant challenge to hyperpolarization based experiments.
